# Health Information Technology (HIT) Adaptation: Refocusing on the Journey to Successful HIT Implementation

**DOI:** 10.2196/medinform.7476

**Published:** 2017-09-07

**Authors:** Po-Yin Yen, Ann Scheck McAlearney, Cynthia J Sieck, Jennifer L Hefner, Timothy R Huerta

**Affiliations:** ^1^ Washington University in St Louis Institute for Informatics St Louis, MO United States; ^2^ Goldfarb School of Nursing, BJC Healthcare St Louis, MO United States; ^3^ The Ohio State University Department of Family Medicine Columbus, OH United States

**Keywords:** health information technology, adaptation, adoption, acceptance

## Abstract

In past years, policies and regulations required hospitals to implement advanced capabilities of certified electronic health records (EHRs) in order to receive financial incentives. This has led to accelerated implementation of health information technologies (HIT) in health care settings. However, measures commonly used to evaluate the success of HIT implementation, such as HIT adoption, technology acceptance, and clinical quality, fail to account for complex sociotechnical variability across contexts and the different trajectories within organizations because of different implementation plans and timelines. We propose a new focus, HIT adaptation, to illuminate factors that facilitate or hinder the connection between use of the EHR and improved quality of care as well as to explore the trajectory of changes in the HIT implementation journey as it is impacted by frequent system upgrades and optimizations. Future research should develop instruments to evaluate the progress of HIT adaptation in both its longitudinal design and its focus on adaptation progress rather than on one cross-sectional outcome, allowing for more generalizability and knowledge transfer.

## Introduction

Health information technology (HIT) is defined as “the application of information processing involving both computer hardware and software that deals with the storage, retrieval, sharing, and use of health care information, data, and knowledge for communication and decision making” [1]. During the past 10 years in the United States, several policies, such as the Health Information Technology for Economic and Clinical Health Act, have led to accelerated HIT adoption and implementation in health care settings, especially implementation of electronic health record (EHR) systems [2,3]. In addition, the Centers for Medicare and Medicaid Services established the EHR incentive program to promote the development of a robust HIT infrastructure, and as part of that effort, released Meaningful Use (MU) criteria in 2010. These criteria require hospitals to implement advanced capabilities of certified EHRs by certain dates in order to receive financial incentives. Other efforts focused on the creation of regional extension centers to facilitate the transition to EHR use through training. MU criteria consist of 3 stages [4]: stage 1, begun in 2011, has a focus on data capture and sharing; stage 2, begun in 2014, aims to improve clinical processes with health information exchange, ePrescription, and patient access; and stage 3, in 2017, was recently replaced by Advanced Care Information [5,6] due to criticism of the MU program [7,8].

Hospitals have been rapidly responding to these new policies and incentives with large-scale implementations of EHRs during the past few years. Adopting new technology requires the redesign of individual and collective workflows and results in changes in both organizational structure and process [9-13]. Yet rapid adoption may hinder the interoperability of the EHR system [14,15]. To facilitate appropriate adoption and use, upgrades, redesign, and optimization are needed, including both minor and major changes in EHR infrastructures, functions, interfaces, and workflows. Further, recent studies have shown that there is a close relationship between the speed of adoption and patient safety concerns of clinicians, both across facilities and within different units [16-18]. EHR implementation could be a distraction from patient care with negative impact on patient outcomes [19] and has mixed association with quality improvement [20,21].

At the same time, studies suggest that unsuccessful implementation of HIT systems could be due to poorly designed HIT, poor use of HIT by clinicians, or socioorganizational factors such as goal conflicts, lack of time, or lack of support from colleagues [22]. However, these studies lack clarity in their measures [23]. This lack of differentiation between technological and human factors thus limits the ability to apply research findings to practice in technology implementation [24].

Given MU regulations, MU requirements have commonly been used as a means to assess HIT implementation success in order to promote essential HIT functionalities [4]. For example, MU stage 2 requires providers to have certain HIT functionalities (eg, computerized provider order entry, personal health record, medication reconciliation) in order to continue to participate in the EHR incentive programs [25]. However, this approach also creates a ceiling effect, hindering the advancement of innovative utilities. While the MU program may accelerate development and implementation of certain key functions, it also slows down other functionalities [26,27]. By focusing on achieving MU, we risk missing the big picture of health care system changes. Therefore, we propose that there is a need to improve our understanding of how to appropriately assess the performance and success of HIT implementation over time to allow us to generalize to other HIT implementation contexts.

### Measuring Health Information Technology Implementation Success

Successful HIT implementation is commonly evaluated using measures such as HIT adoption, technology acceptance, and clinical quality. Yet this disparate array of measures fails to account for complex sociotechnical interactions, variability across contexts, and the different trajectories within organizations that exist because of different implementation plans and timelines. Appropriate measurement of HIT implementation thus needs to take into account this variability across organizations and over time but at the same time enable us to generalize the variation across HIT implementation studies in order to inform practice. As a result, the issue of consistent measurement becomes increasingly significant.

Current measures that exist in the literature include HIT adoption, HIT acceptance, and clinical quality measures (CQMs). The first common measure, HIT adoption, is defined by the EHR MU stages outlined by the Office of the National Coordinator and measures the rate of health care systems having chosen to invest resources toward EHR implementation. It is commonly reported as an adoption rate to reflect the percentage of health care organizations with specific EHR functionalities or capabilities that are meaningful for patient care. In 2013, 59% of hospitals reported at least a basic EHR system, but only 5.1% could meet the MU stage 2 criteria [2]. The expectation is that more meaningful use of an EHR system will ultimately result in improved care and more empowered clinicians. In addition, the Healthcare Information and Management Systems Society (HIMSS) measures EHR adoption through the Electronic Medical Record Adoption Model (EMRAM), which categorizes EHR capabilities into an 8-stage scale from stage 0 to stage 7 [28]. In 2015, HIMSS Analytics’ Annual Study reported that 27% of hospitals are at stage 6 or above. Although it is helpful to recognize the EHR capabilities across organizations in the nation, it is unclear whether those functions are fully used by clinicians.

The second approach to measuring implementation success involves HIT acceptance, the extent of individual commitment to use the technology [29-33]. When assessing individual user acceptance, the technology acceptance model (TAM) [34,35] is a commonly applied and useful model, albeit with limitations [36]. TAM’s predictive power in health care is lower than what has been found in other domains [24], and some recommend that the TAM should be integrated with other adoption theories [36], particularly those that include variables related to both human and social change processes [24].

CQMs [37] are another common metric used to assess the success of HIT [38]. However, HIT implementation appears to have little impact on care quality whether measured by patient mortality, adverse drug events, or readmission rates [39]. Although CQMs are helpful for assessing the extent to which HIT can be used to monitor the quality of health care services provided, this approach to measurement does not take into account organizational or human factors that could impact HIT implementation.

Measuring HIT adoption and acceptance alone provides only a limited understanding of HIT success. Both HIT adoption rates and TAM are helpful to understand the status of HIT implementation and acceptance, but they do not inform a strategic plan for promoting successful HIT implementation in a health care organization. CQM as a proxy for HIT success also fails to take into account the organizational context of implementation. In short, as HIT implementation is a process, not an outcome, understanding implementation success requires consideration of the sociotechnical environment in which it takes place.

### Sociotechnical Theory: Improving Our Understanding of Health Information Technology Implementation

Sociotechnical theory positions people-focused (socio) elements, organizational and human, and information technology elements (technical) as interdependent parts of a system that cannot be studied in isolation and therefore must be evaluated together [40]. Sociotechnical theory has been discussed as a theoretical framework that is responsive to the tenets of complex adaptive systems (CAS) [41-44]. When viewed in concert, these 2 theoretical approaches support that interdependent interactions between people and technology within the workplace have both direct impacts, in the classical cause and effect sense, and impacts through feedback, where these same people and technology attenuate, strengthen, distort, halt, or change valence over time [41,43,45].

Current sociotechnical evaluations involve assessing both the technology and the social contexts where the technology is implemented. A systematic review conducted on EHR implementations revealed that sociotechnical factors complicate HIT deployments [46]. Technical features of HIT interact with the social features of a health care work environment. Further, it has been demonstrated that the quality of the implementation process is just as important as the features and capabilities of the system being implemented [47-49].

We suggest grounding the theoretical framing of CAS that refers to adaptiveness as “the ability of components of a CAS to change their behavior as a result of interactions with the other components and the surroundings” [41]. In shifting the concept of adoption to adaptation, we frame sociotechnological change as occurring over time with system response characterized as the adaptiveness of a health care organization in the context of changes to HIT implementation [42,44]. For example, technical features are not static; rather they frequently change over time as new versions of the software are promulgated. As such, adoption is not an end state; it is the application of an arbitrary end point to facilitate our understanding. From that perspective, understanding the adaptiveness, or HIT adaptation in this process, is thus significant in our understanding of HIT implementation success [50].

### Health Information Technology Adaptation

Although sociotechnical theory and CAS have been used to explain complexity in health care [51], little has been discussed that uses adaptation as a measure to evaluate the success of HIT implementation over time. We thus propose a new focus: adaptation. Adaptation is conceptualized as “a process of modifying existing conditions in an effort to achieve alignment” [52] involving workflow redesign, user trainings, and technology maintenance [53]. In the context of HIT implementation, refocusing from adoption and acceptance to adaptation illuminates factors that facilitate or hinder the connection between use of the EHR and improved quality of care. Further, by shifting to adaptation, we refocus the question of HIT adoption to one that explores the trajectory of change as an explicit component of the way we measure these issues. Table 1 presents the definitions of adoption, adaptation, and acceptance as differentiated by Cooper and Zmud [53].

**Table 1:** Definitions of adoption, adaptation, and acceptance [54].

Concept	Definition
Adoption	A decision is reached to invest resources to accommodate the implementation effort.
Adaptation	The innovation is developed, installed, and maintained. Procedures are developed and revised. Members are trained both in the new procedures and in the innovation.
Acceptance	Organizational members are induced to commit to the innovation’s usage.

MU criteria [54-56] and CQMs can be seen as verification and validation steps, respectively, for HIT implementation. In product or system design, evaluation is commonly done via verification and validation. Verification serves as quality control to assess whether a system is in compliance with regulations and specifications. On the other hand, validation is a quality assurance process that establishes evidence to ensure a system accomplishes what was intended. However, no measures have been proposed to assess HIT implementation performance between the steps of system verification and validation. We suggest that this period encompasses the HIT adaptation process, requiring its own measurement approach.

**Figure 1:**
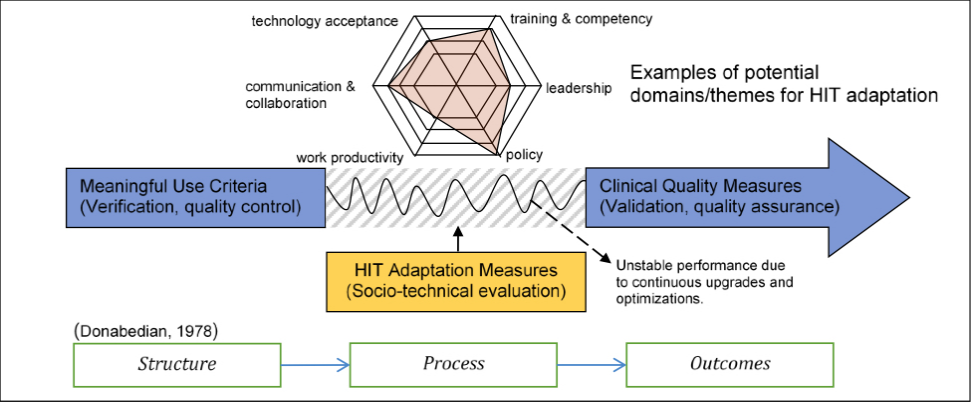
Health information technology adaptation measures as the process evaluation.

In Figure 1, we illustrate the current knowledge gap between meeting the MU criteria and achieving CQMs, linking this conceptually to Donabedian’s well-known structure-process-outcomes model, a quality assessment model presented to evaluate health services outcomes [57]. Considering HIT implementation in the context of the Donabedian model, structure refers to HIT resources, which are determined by MU criteria; process refers to clinicians’ use or adaptation of HIT for their use; and outcomes refer to the effects of using HIT for the delivery of health care, as measured by CQMs. In practice, the HIT implementation journey will be impacted by frequent system upgrades and optimizations, leading to performance variability throughout the process. However, by including considerations of sociotechnical factors such as technology acceptance, communication and collaboration, work productivity, training and competency, leadership, and policy, the progress of HIT adaptation could be appropriately assessed.

### Theoretical Frameworks to Study Health Information Technology Adaptation

**Figure 2:**
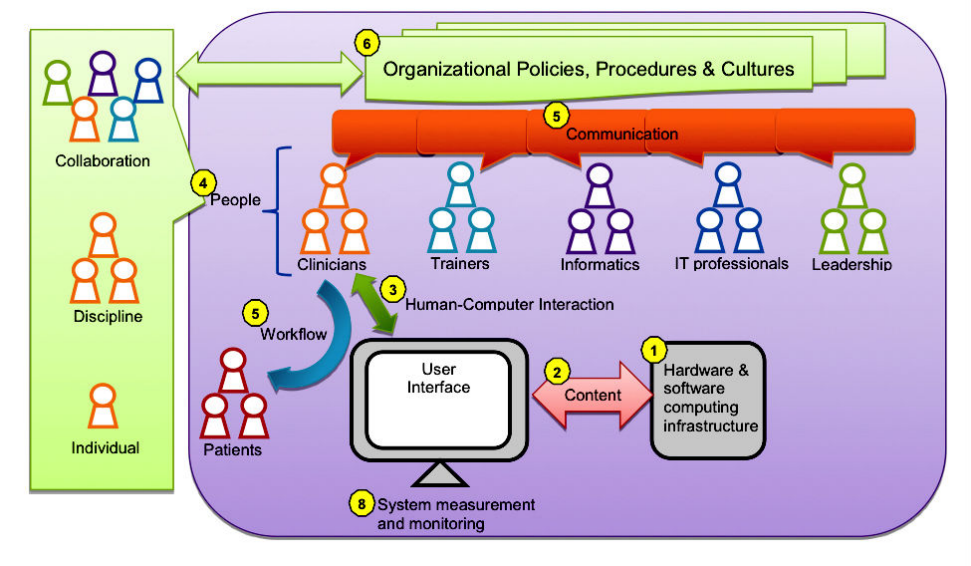
Adapted from the sociotechnical model [43].

Two theoretical frameworks provide guidance for HIT adaptation research: the information technology (IT) implementation framework [58] and a new sociotechnical model [42]. First, the IT implementation framework [58] suggests that (1) IT use is complex, multidimensional, and influenced by a variety of factors at individual and organizational levels and (2) success in achieving change is enhanced by active participation of members from the target user group [58]. The new sociotechnical model [42] now aims to study HIT in complex adaptive health care systems and suggests investigating 8 dimensions: (1) hardware and software computing infrastructure; (2) clinical content; (3) human-computer interface; (4) people; (5) workflow and communication; (6) internal organizational policies, procedures, and culture; (7) external rules, regulations, and pressures; and (8) system measurement and monitoring [42]. Figure 2 illustrates our adapted model from the new sociotechnical model [42]. We do not include the seventh dimension, “external rules, regulations, and pressures,” as we focus on factors within the organization.

### Recommendations for Future Health Information Technology Adaptation Research

We propose that HIT adaptation research should deploy multilevel and multidimensional evaluation to understand the HIT adaptation progress, drawing from both of these foundational theories. Specifically, HIT adaptation research should focus on developing fundamental and multidimensional facts that can inform the progress of HIT adaptation. Below we describe 4 directions that can drive future HIT adaptation research.

#### Develop Appropriate Process Measures

While the outcome measures (HIT adoption rate, acceptance, and CQMs) have been established, there is a need to develop process measures from individual and organizational perspectives and include multidimensional measures of adaptation to EHRs. These measures will need to incorporate factors such as communication channels, cultural conflict, interdisciplinary team dynamics, user satisfaction, work productivity, cost, and quality [38,59,60].

#### Consider the Culture and Context in Which Health Information Technology Is Implemented

Most HIT adoption or acceptance studies have used individuals or hospitals as the unit of analysis [39,61,62]. These findings are informative for identifying associated individual perceptions and experiences as well as hospital demographics. However, additional factors such as the culture of a discipline or a department, the interprofessional or multidisciplinary communication within or across departments, the training received, and workflow at the department level have not been discussed. In particular, while social support has been identified as one of the key factors for acceptance [63,64], no studies have been conducted at the department or unit level to study this factor.

#### Standardize the Definition and Methods for Sociotechnical Studies

Implementing a new technology into a complex environment is often disruptive, particularly in health care. Sociotechnical evaluations of HIT implementations are supported in both theory and empirically; however, little guidance exists in terms of how to conduct a sociotechnical evaluation [65]. Challenges in conducting sociotechnical evaluations include a lack of agreement on the components of the sociotechnical system, possible study designs, and data analysis strategies which may give light to both practical and conceptual challenges [65].

#### Study Adaptation Longitudinally and Multidimensionally

Processes are more important to study than outcomes because studying processes allows for generalizability and knowledge transfer beyond the clinical setting where the research was conducted [65]. Future studies need to employ longitudinal study designs with multiple data time periods to establish causal relationships [32,66,67]. In addition, the HIT evaluation toolkit proposed by the Agency for Healthcare Research and Quality emphasizes the advantages of conducting mixed methods studies to provide important dimensions in an evaluation study [68]. Thus, future HIT research studies should be designed as mixed methods sociotechnical evaluations focused on exploring the dynamic relationship between technology and social factors over time [65].

### Conclusion

Measuring HIT adaptation can provide a more thorough understanding of the connection between HIT use and health care outcomes. Our ability to advance our understanding is predicated on good evaluation models, notably in the area of a health organization’s overall performance. As the sociotechnical environment remains a confounding problem influencing our understanding of the generalizability of research findings about HIT implementation success, there is a need to integrate issues exacerbated by workarounds, poorly designed interfaces, suboptimal functionality, and the sheer complexity of systems that contribute to HIT adoption issues as well as consider the idiosyncrasies across contexts. However, existing evaluation models are not supportive of a greater understanding of the phenomenon itself. This paper is therefore presented to provide a new perspective to shift the focus from adoption to adaptation. Future research should develop instruments to evaluate the progress of HIT adaptation in both its longitudinal design and its focus on adaptation progress rather than on a single outcome, allowing for more generalizability and knowledge transfer.

## Abbreviations

CAS: complex adaptive system
CQM: clinical quality measure
EMRAM: electronic medical record adoption model
HER: electronic health record
HIMSS: Healthcare Information and Management Systems Society
HIT: health information technology
IT: information technology
MU: Meaningful Use
TAM: technology acceptance model
